# Shelter-in-Place Policies, Proximity, and Changes in Caregiving for Older Adults During the COVID-19 Pandemic

**DOI:** 10.1093/geroni/igaf122.804

**Published:** 2025-12-31

**Authors:** Lei Chen, Joanne Spetz

**Affiliations:** University of California, San Francisco, San Francisco, California, United States; University of California, San Francisco, San Francisco, California, United States

## Abstract

During the COVID-19 pandemic, the dynamics of family support for older adults living with disabilities were disrupted due to “Shelter-in-Place (SIP)” orders. This study examined the impact of SIP policies on caregiving changes and how these changes were related to family members’ geographic proximity. This study used the National Health and Aging Trends Survey (NHATS) round 10 data, COVID-19 geographic data, and restricted COVID-19 data files. We added published data about SIP policies at the county level. The study sample was NHATS respondents who needed assistance with activities of daily living (ADL) or instrumental activities of daily living (IADL), and who did not live with a spouse (N = 512). We estimated weighted multivariate logistic regression models and estimated separately by dementia status and ADL/IADL needs. 55.2% reported no change in receiving help, 36.7% reported receiving less help, and 8.1% reported receiving more help during COVID than before. Compared to those who did not have a change in help during COVID, a person living in a place with a higher percentage of SIP days was less likely to receive more help. No significant changes were found as stratified by dementia status and ADL/IADL needs. No significant associations were found between proximity and changes in caregiving. It is important to address caregiving needs of older adults with disabilities who do not live with a spouse during public health emergencies and to examine how disruptions caused by policies impact the provision of help for people who rely on family support.

